# Effects of embryonic day and *in ovo* feeding of *β*‑hydroxy-*β*-methylbutyrate on hatching performance, organ development, and jejunal morphology in Japanese quail

**DOI:** 10.1016/j.psj.2026.107731

**Published:** 2026-09-05

**Authors:** Ji Won Shin, Geun Yong Park, Yeong Bin Kim, Ju Yeong Park, Jin Ho Jeong, Seung Yun Lee, Jong Hyuk Kim

**Affiliations:** a Department of Animal Science, Chungbuk National University, Cheongju, 28644, Republic of Korea; b Division of Animal Science, Institute of Agriculture & Life Science, Gyeongsang National University, Jinju, 52828, Republic of Korea

**Keywords:** *β*-hydroxy-*β*-methylbutyrate, Hatchability, *In ovo* feeding, Japanese quail, Jejunal morphology

## Abstract

The objective of this study was to investigate the effects of embryonic day and *in ovo* feeding of *β*‑hydroxy-*β*-methylbutyrate (HMB) on hatching performance, organ development, and jejunal morphology in Japanese quail. A total of 520 fertile eggs were assigned to a 2 × 2 factorial arrangement with two embryonic days of *in ovo* feeding, embryonic day 7 (E7) and embryonic day 11 (E11), and two *in ovo* feeding treatments, 1× PBS and 1% HMB. Each treatment included 10 replicates with 13 eggs per replicate, and 0.05 mL of the assigned solution was injected into the air cell. The results indicated a significant interaction (*P* < 0.05) between the embryonic day of *in ovo* feeding and *in ovo* feeding treatment for hatching performance and hatchability. On E7, the HMB group showed greater (*P* < 0.05) body weight (BW) and chick yield, and a shorter (*P* < 0.05) hatch window than the PBS group. On E11, the HMB group showed decreased (*P* < 0.05) post-pipping mortality compared to the PBS group. Regarding the main effect of the embryonic day of *in ovo* feeding, the E11 groups showed increased (*P* < 0.05) relative liver and intestinal weights compared to the E7 groups. Regarding the main effect of *in ovo* feeding treatment, *in ovo* feeding of HMB increased (*P* < 0.05) hatch of set, hatch of fertile, BW, chick yield, radius, villus height, villus width, and villus height-to-crypt depth ratio compared with *in ovo* feeding of PBS. *In ovo* feeding of HMB decreased (*P* < 0.05) the hatch window and late embryo mortality compared with *in ovo* feeding with PBS. In conclusion, *in ovo* feeding of HMB may improve hatching performance, reduce late embryonic mortality, and support jejunal development in Japanese quail, with responses partly depending on the embryonic day of administration.

## Introduction

The Japanese quail (*Coturnix japonica*) has been widely used both as a minor commercial poultry species and as an experimental avian model because of its short incubation period, early sexual maturity, and relatively accessible embryos (Huss et al., 2008; Ainsworth et al., 2010). Embryonic development in the Japanese quail generally parallels that of the chicken during early development but proceeds at a faster rate, making this species useful for evaluating *in ovo* nutritional interventions (Ainsworth et al., 2010). In commercial production, hatching performance and hatchability are key determinants of reproductive efficiency and are closely related to embryonic viability and developmental uniformity (Tona et al., 2003). The final stage of incubation is metabolically demanding because the embryo transitions from chorioallantoic to pulmonary respiration and completes internal and external pipping (Visschedijk, 1968a; Decuypere and Bruggeman, 2007). During this period, the embryo faces increasing metabolic and developmental demands while relying on the finite nutrient reserves within the egg. In particular, carbohydrate availability may become limiting during the late embryonic period because glucose and glycogen are important energy substrates for pipping and the transition to pulmonary respiration, and depletion of these reserves may constrain the energy available for successful hatching (Christensen et al., 2001; Pulikanti et al., 2010). These physiological constraints have encouraged interest in nutritional strategies that can support or modulate embryonic development before hatching (Givisiez et al., 2020).

The *in ovo* feeding technique, defined as the controlled delivery of a small volume of nutrients or a bioactive solution into an incubating egg, has been proposed as a strategy to modulate embryonic development before hatching (Uni and Ferket, 2004). In chickens and turkeys, *in ovo* feeding has been reported to influence hatchability, hatching body weight (BW), embryonic energy status, and intestinal mucosal development. However, responses can vary depending on the compound, dose, injection route, and embryonic stage of administration (Uni et al., 2005; Foye et al., 2006). As intestinal maturation accelerates during the final stages of incubation, including villus development and crypt formation, *in ovo* feeding has also been investigated as a means of supporting intestinal structural development before the transition to post-hatch feed utilization (Uni et al., 1998; Tako et al., 2004; Smirnov et al., 2006).

Among candidate bioactive compounds for *in ovo* feeding, *β*‑hydroxy-*β*-methylbutyrate (HMB), a metabolite of the branched-chain amino acid leucine, has received attention because of its reported involvement in protein metabolism and tissue development (Nissen and Abumrad, 1997). In poultry, *in ovo* feeding solutions containing HMB have been reported to affect hatchability, hatching weight, and intestinal development in turkeys and broilers (Tako et al., 2004; Foye et al., 2007; Ma et al., 2020). Dietary HMB has also been associated with changes in bone parameters, intestinal morphology, and muscle-related traits in laying hens (Tomaszewska et al., 2024). However, several previous *in ovo* studies used combined nutrient solutions; therefore, the observed responses cannot be attributed solely to HMB.

The embryonic stage at administration may be an important factor influencing the response to *in ovo* feeding of HMB because developmental priorities differ across incubation (Kpodo and Proszkowiec-Weglarz, 2023). Earlier stages are characterized by active tissue differentiation and skeletal development, whereas later stages are more closely associated with preparation for pipping, hatching, and late embryonic intestinal maturation (Givisiez et al., 2020). In Japanese quail, developmental events are compressed relative to those in broiler chickens, and long-bone development is already underway during the first half of incubation (Nakane and Tsudzuki, 1999; Ainsworth et al., 2010). Embryonic day 7 (E7) represents a relatively early developmental window during which tissue differentiation and musculoskeletal development, including ongoing skeletal formation, remain active (Nakane and Tsudzuki, 1999; Ahmed and Soliman, 2013), whereas embryonic day 11 (E11) represents a later developmental window that is closer to late embryonic intestinal maturation and the physiological preparation for pipping and hatching in Japanese quail (Ainsworth et al., 2010; Givisiez et al., 2020). Therefore, the response to *in ovo* feeding of HMB may differ depending on whether it is administered at E7 or E11. However, it remains unclear whether Japanese quail embryos respond differently to HMB according to the embryonic stage of administration. Therefore, we hypothesized that *in ovo* feeding of HMB would influence hatching performance, hatchability, morphometric parameters, relative organ and muscle weights, and jejunal morphology in Japanese quail, and that these responses would depend on the embryonic stage at administration.

Accordingly, the objective of this study was to investigate the effects of the embryonic day of *in ovo* feeding of HMB treatment on hatching performance, hatchability, morphometric parameters, relative organ and muscle weights, and jejunal morphology in Japanese quail.

## Materials and methods

### Eggs and incubation

The study protocol for this experiment was approved by the Institutional Animal Care and Use Committee of Chungbuk National University (CBNUA-25-0085-01). A total of 520 hatching eggs were obtained from 16-week-old Japanese quail at a local commercial hatchery (Geumwang Farm, Eumseong, Republic of Korea). The eggs were allotted to four treatments in a 2 × 2 factorial arrangement, consisting of two *in ovo* feeding days and two *in ovo* feeding treatments, with 10 replicates per treatment and 13 eggs per replicate. The main effects were the embryonic day of *in ovo* feeding and the *in ovo* feeding treatment. The *in ovo* feeding procedure was performed on E7 and E11, and either 1× PBS or 1% HMB was administered into the eggs. All fertile eggs were incubated under recommended conditions, with temperature maintained at 37.5 **±** 0.2°C throughout the incubation period. Relative humidity was set at 60 **±** 3.0% during 0 to 13 days and increased to 70 **±** 3.0% during 14 to 18 days of incubation. Before incubation, the incubator was pre-warmed at 25°C and 60% relative humidity for 3 h. Each egg was then individually weighed and labeled before incubation. Fertile eggs were randomly placed in an automatic incubator (Rcom MARU Deluxe Max 200; Autoelex Co., Ltd., Gimhae, Republic of Korea) under optimal temperature, humidity, and ventilation conditions. Egg turning was automatically programmed at 2-h intervals for a total of 12 times per day, and turning was discontinued after 14 days of incubation.

### Preparation of injection solutions and in ovo feeding procedure

The injection solution was prepared as a 1% (w/v) solution by dissolving 1 g of HMB powder (Nutricost, Vineyard, UT, USA) in 100 mL of PBS (LB004-01, Welgene Inc., Gyeongsan, Republic of Korea). The solution preparation and *in ovo* feeding procedures were conducted according to the method described by Yu et al. (2018) and Park et al. (2026a), with minor modifications. The *in ovo* feeding was conducted on two embryonic days, days 7 and 11. All eggs assigned to the respective experimental treatments were subjected to the injection procedure, and fertility was determined after the injection procedure. Prior to injection, the shell surface at the injection site was disinfected with 70% ethanol. A small hole was created at the blunt end of the egg using a 31-gauge, 8-mm sterile needle (insulin syringe; Sungshim Medical Co., Ltd., Bucheon, Republic of Korea) and 0.05 mL of the prepared solution was injected into the air cell. The air cell was selected as the injection site because it provides a relatively minimally invasive route for administration during early embryonic development and has previously been used for HMB administration in poultry embryos (Dean et al., 2016; Kermanshahi et al., 2017). Compounds administered into the air cell may subsequently reach the embryo through transfer associated with the developing chorioallantoic membrane (Siwek et al., 2018). However, the actual uptake of HMB by the embryo was not directly measured in the present study. The hole was sealed using a clear nail polish (Ringring Solid Clear; Organic Farm Korea, Hwaseong, Republic of Korea). The needle was replaced with a new sterile needle after each use during the injection procedure.

### Hatching performance

Measurements were conducted according to the method described by Muyyarikkandy et al. (2023) and Park et al. (2026b), with minor modifications. Initial egg weight was measured prior to incubation using an electronic scale (HS-1000A; Hansung Co., Ltd., Seoul, Republic of Korea). Hatching performance was evaluated based on hatch of set, hatch of fertile, BW at hatch, hatch window, and chick yield. The BW and hatching time of each chick were recorded immediately after hatching. Hatch of set was calculated as the percentage of chicks hatched from the total number of eggs set, whereas hatch of fertile was calculated as the percentage of chicks hatched from the fertile eggs. The hatch window was defined as the time interval between the first and the last chick hatched within each replicate. Chick yield was calculated as the percentage obtained by dividing the average hatched chick weight by the average fertile egg weight.

### Hatchability

Hatchability-related percentages were calculated on a fertile egg basis within each replicate. The fertility of the set eggs represents the proportion of fertile eggs relative to the total number of eggs set. It was calculated by dividing the number of fertile eggs by the total number of eggs set. The contaminated egg rate represents the proportion of contaminated eggs among the fertile eggs. It was calculated by dividing the number of contaminated eggs by the number of fertile eggs. Embryo mortality was classified into early (1 to 3 d), middle (4 to 10 d), and late (11 to 17 d) mortality according to the stage of embryonic development. Each embryonic mortality rate was calculated by dividing the number of dead embryos in each period by the number of fertile eggs. Post-pipping mortality represents the percentage of chicks that died after pipping, and it was calculated by dividing the number of post-pipping deaths by the number of fertile eggs. Culled chicks represent the percentage of chicks exhibiting abnormalities, such as unhealed navels, skin lesions, beak deformities, and leg abnormalities. The number of abnormal chicks was divided by the number of fertile eggs to calculate this percentage.

### Morphometry

Chicks were euthanized immediately after hatching using CO_2_, and morphometric indices as well as the weights of the breast muscle, leg muscle, and liver were subsequently determined. These tissues were excised and individually weighed using a digital scale (HS1000A; Hansung Co., Ltd., Seoul, Republic of Korea). Morphometric assessments included measurements of the tibia, radius, small intestine, and chick body lengths. The tibia and radius were measured using a flexible measuring tape after the surrounding muscles were removed from the legs and wings, respectively. The small intestine was extended without excessive tension and measured using a flexible measuring tape to minimize tissue distortion. Chick body length was measured from the tip of the beak to the base of the middle toe using a flexible measuring tape. All measurements were conducted by a single trained individual to ensure consistency and minimize inter-observer variation.

### Relative organ and muscle weights

The relative organ and muscle weights of the heart, liver, gizzard, proventriculus, intestine, breast, and thigh muscles of Japanese quail chicks were determined using a digital scale (HS1000A; Hansung Co., Ltd., Seoul, Republic of Korea) and expressed relative to BW.

### Jejunal morphology

A jejunal tissue segment approximately 3 cm in length was collected from the midpoint of the jejunum and fixed in 10% buffered formalin. The fixed tissues were embedded in paraffin, sectioned at a thickness of 4 µm, and stained with hematoxylin and eosin for histological evaluation. The stained sections were examined using a microscope (OS-370DVM; Osun Hitech, Goyang, Republic of Korea). Villus height (VH) was measured from the base of the villus to its tip, whereas crypt depth (CD) was measured from the base of the villus to the base of the crypt. Villus width (VW) was measured at the base of each villus. VH, VW, CD, and VH:CD ratio were calculated as the mean of 20 measurements for each parameter. The detailed procedures followed the method previously described by Kim et al. (2024).

### Statistical analysis

All data were analyzed by two-way ANOVA in a 2 × 2 factorial arrangement using the PROC MIXED procedure of SAS (SAS Institute Inc., Cary, NC, USA). Each replicate was considered an experimental unit for all measurements. Outliers were assessed using the PROC UNIVARIATE procedure of SAS and by inspecting studentized residuals and residual-versus-predicted-value plots generated using PROC GLM. Observations showing marked deviations were further evaluated for biological plausibility before exclusion. No predefined numerical threshold was used for outlier identification, and observations considered outliers based on both statistical diagnostics and assessment of biological plausibility were excluded from the statistical analysis. The fixed effects in the model included the embryonic day of *in ovo* feeding, *in ovo* treatment, and their interaction. No random effects were specified in the model. Percentage data were analyzed using the same statistical model without transformation after examination of their distributions and model residuals. Least-squares means were estimated using the LSMEANS statement, and pairwise comparisons among least-squares means were performed using the PDIFF option without adjustment for multiple comparisons. Data are presented as least-squares means ± SEM. Statistical significance was set at *P* < 0.05.

## Results

### Hatching performance

Initial egg weight was not affected by embryonic day, *in ovo* treatment, or their interaction (Table 1). For hatching performance, a significant interaction (*P* < 0.05) between *in ovo* feeding treatment and injection day was observed for BW, hatch window, and chick yield in Japanese quail. On E7, the HMB group showed a greater BW and chick yield, with a reduced hatch window, compared with the PBS group. On E11, in contrast, no differences were observed between the treatments. Regarding the main effects of embryonic day of *in ovo* feeding, there were no differences in hatching performance between embryonic days. However, the HMB groups showed greater (*P* < 0.05) hatch of set, hatch of fertile, BW, and chick yield than the PBS groups.

**Table 1:** Effects of *in ovo* feeding of *β*-hydroxy-*β*-methylbutyrate (HMB) on initial egg weight and hatching performance in Japanese quail.^1^

Items	*In ovo* feeding	Egg weight, g	Hatch of set^2^, %	Hatch of fertile^3^, %	BW, g	Hatch window^4^, h	Chick yield^5^, %
Embryonic day 7	PBS	11.16	63.08	67.61	8.31^c^	18.20^a^	74.49^c^
	HMB	11.15	72.65	76.72	8.57^a^	5.66^b^	76.85^a^
Embryonic day 11	PBS	11.15	71.54	74.55	8.41^bc^	12.01^ab^	75.41^bc^
	HMB	11.15	76.92	82.01	8.47^ab^	13.49^ab^	75.98^ab^
SEM (n = 10)		0.010	3.516	3.613	0.050	2.814	0.439
Main effect							
Embryonic day							
7		11.16	67.86	72.16	8.44	11.93	75.67
11		11.15	74.23	78.28	8.44	12.75	75.70
SEM (n = 20)		0.007	2.423	2.490	0.034	1.939	0.303
Treatment							
PBS		11.16	67.31	71.08	8.36	15.11	74.95
HMB		11.15	74.79	79.36	8.52	9.58	76.41
SEM (n = 20)		0.007	2.486	2.554	0.035	1.990	0.311
*P*-value							
Embryonic day		0.575	0.072	0.091	0.978	0.768	0.950
Treatment		0.575	0.036	0.025	0.003	0.052	0.002
Embryonic × Treatment		0.959	0.545	0.817	0.048	0.015	0.044

a-c Means with different superscripts within the same row indicate a significant difference at *P* < 0.05.

1 Each value represents the mean of 10 replicates per each *in ovo* feeding of treatment.

2 Hatch of set = (number of eggs hatched / number of eggs set) × 100.

3 Hatch of fertile = (number of eggs hatched / number of fertile eggs) × 100.

4 Hatch window = time of the last hatched chick - time of the first hatched chick.

5 Chick yield = (average hatched chick weight / average fertile egg weight) × 100.

### Hatchability

There was an interaction (*P* < 0.05) between the embryonic day of *in ovo* feeding and *in ovo* feeding treatment for post-pipping mortality (Table 2). On E11, the HMB decreased post-pipping mortality compared with PBS group, whereas no differences between *in ovo* feeding treatments were observed on E7. As the main effect of *in ovo* feeding treatment, the HMB groups showed lower (*P* < 0.05) late embryo mortality than the PBS group.

**Table 2:** Effects of *in ovo* feeding of *β*-hydroxy-*β*-methylbutyrate (HMB) on hatchability in Japanese quail.^1^

Items	*In ovo* feeding	Fertility of set eggs^2^, %	Contaminated eggs^3^, %	Embryo mortality^4^, %			Post-pipping mortality^5^, %	Culls^6^, %
				Early	Middle	Late		
Embryonic day 7	PBS	92.31	1.60	10.80	5.25	15.03	1.67^ab^	5.11
	HMB	93.08	0.83	11.44	4.27	7.42	3.42^ab^	2.54
Embryonic day 11	PBS	96.15	1.60	6.28	7.12	10.45	4.81^a^	3.21
	HMB	93.85	2.44	8.16	4.81	4.11	0.00^b^	4.95
SEM (n = 10)		2.183	1.063	2.357	1.944	2.383	1.420	1.345
Main effect								
Embryonic day								
7		92.69	1.22	11.12	4.76	11.23	2.54	3.83
11		95.00	2.02	7.22	5.96	7.28	2.40	4.08
SEM (n = 20)		1.544	0.752	1.666	1.375	1.685	1.004	0.951
Treatment								
PBS		94.23	1.60	8.54	6.19	12.74	3.24	4.16
HMB		93.46	1.64	9.80	4.54	5.77	1.71	3.74
SEM (n = 20)		1.544	0.752	1.666	1.375	1.685	1.004	0.951
*P*-value								
Embryonic day		0.298	0.456	0.107	0.541	0.107	0.922	0.854
Treatment		0.727	0.976	0.597	0.402	0.006	0.290	0.759
Embryonic × Treatment		0.486	0.456	0.792	0.736	0.790	0.027	0.117

a,b Means with different superscripts within the same row indicate a significant difference at *P* < 0.05.

1 Each value represents the mean of 10 replicates per each *in ovo* feeding of treatment.

2 Fertility of set eggs = (number of fertile eggs / number of eggs set) × 100.

3 Contaminated eggs = (number of contaminated eggs / number of fertile eggs) × 100.

4 Early (1 to 3 d), middle (4 to 10 d) or late mortality (11 to 17 d) calculated based upon the number of fertility eggs.

5 Post-pipping mortality = (number of chicks that died after pipping / number of fertile eggs) × 100.

6 Culled chicks (unhealed navel, skin lesions, deformed beak or abnormal conformation of legs) calculated based upon the number of fertile eggs.

### Morphometry

No significant interactions between the embryonic day of *in ovo* feeding and *in ovo* feeding treatment were observed for the morphometric parameters (Table 3). In terms of the main effect of embryonic day of *in ovo* feeding, there were no differences in morphometry between the injection days. However, regarding the main effect of *in ovo* feeding treatment, relative radius was greater (*P* < 0.05) in the HMB groups than in the PBS groups.

**Table 3:** Effects of *in ovo* feeding of *β*-hydroxy-*β*-methylbutyrate (HMB) on morphometry in Japanese quail.^1^

Items	*In ovo* feeding	Tibia^2^, %	Radius^3^, %	Intestine, cm	Chick length^4^, cm
Embryonic day 7	PBS	18.99	7.67	13.94	9.90
	HMB	18.68	8.12	14.36	10.26
Embryonic day 11	PBS	19.42	7.48	15.30	10.17
	HMB	19.78	8.17	14.53	10.06
SEM (n = 10)		0.546	0.269	0.401	0.206
Main effect					
Embryonic day					
7		18.84	7.90	14.15	10.08
11		19.60	7.82	14.92	10.12
SEM (n = 20)		0.386	0.190	0.284	0.145
Treatment					
PBS		19.21	7.58	14.62	10.04
HMB		19.23	8.14	14.45	10.16
SEM (n = 20)		0.386	0.190	0.284	0.145
*P*-value					
Embryonic day		0.170	0.785	0.065	0.866
Treatment		0.971	0.043	0.665	0.547
Embryonic × Treatment		0.540	0.668	0.147	0.261

1 Each value represents the mean of 10 replicates per each *in ovo* feeding of treatment.

2 Tibia = (tibia length / chick length) × 100.

3 Radius = (radius length / chick length) × 100.

4 Chick length = measured from the tip of the middle toe to the tip of the beak.

### Relative organ and muscle weights

A significant interaction (*P* < 0.05) between the embryonic day of *in ovo* feeding and *in ovo* feeding treatment was observed for relative heart weight (Table 4). On E7, the HMB group had a significantly lower (*P* < 0.05) relative heart weight than the PBS group. However, no differences were identified between the treatments on E11. As the main effects of the embryonic day of *in ovo* feeding, the E11 groups had greater (*P* < 0.05) relative liver and intestinal weights than the E7 groups. In addition, the relative liver, proventriculus, and intestinal weights were lower (*P* < 0.05) in the HMB groups than in the PBS groups.

**Table 4:** Effects of *in ovo* feeding of *β*-hydroxy-*β*-methylbutyrate (HMB) on relative organ and muscle weights in Japanese quail.^1^

Items^2^	*In ovo* feeding	Heart, %	Liver, %	Gizzard, %	Proventriculus, %	Intestine, %	Breast, %	Thigh, %
Embryonic day 7	PBS	1.38^a^	2.86	4.30	1.24	3.96	2.30	2.12
	HMB	1.16^b^	2.62	4.08	1.09	3.64	2.41	2.06
Embryonic day 11	PBS	1.25^ab^	3.39	4.98	1.46	4.96	2.45	2.07
	HMB	1.29^ab^	3.02	4.10	1.16	4.07	2.32	2.00
SEM (n = 10)		0.060	0.116	0.283	0.087	0.223	0.188	0.132
Main effect								
Embryonic day								
7		1.27	2.74	4.19	1.16	3.80	2.36	2.09
11		1.27	3.21	4.54	1.30	4.52	2.38	2.04
SEM (n = 20)		0.043	0.082	0.200	0.061	0.158	0.133	0.093
Treatment								
PBS		1.31	3.13	4.64	1.35	4.46	2.38	2.10
HMB		1.23	2.82	4.09	1.12	3.86	2.36	2.03
SEM (n = 20)		0.043	0.082	0.200	0.061	0.158	0.133	0.093
*P*-value								
Embryonic day		0.956	<0.001	0.227	0.097	0.003	0.888	0.703
Treatment		0.162	0.012	0.058	0.013	0.010	0.950	0.613
Embryonic × Treatment		0.041	0.582	0.246	0.373	0.211	0.527	0.983

a,b Means with different superscripts within the same row indicate a significant difference at *P* < 0.05.

1 Each value represents the mean of 10 replicates per each *in ovo* feeding of treatment.

2 The relative organ weight was expressed as a percentage of BW.

### Jejunal morphology

No significant interactions between the embryonic day of *in ovo* feeding and *in ovo* feeding treatment were observed for jejunal morphology measurements (Table 5). In terms of the main effects of the embryonic day of *in ovo* feeding, none of the jejunal morphology measurements differed among the injection days. In contrast, the HMB groups showed increased (*P* < 0.05) VH, VW, and VH:CD ratios compared with the PBS groups.

**Table 5:** Effects of *in ovo* feeding of *β*-hydroxy-*β*-methylbutyrate (HMB) on jejunal morphology in Japanese quail.^1^

Items	*In ovo* feeding	VH, μm	CD, μm	VW, μm	VH:CD
Embryonic day 7	PBS	170	26.2	31.7	6.41
	HMB	189	25.8	35.2	7.02
Embryonic day 11	PBS	170	26.5	32.3	6.72
	HMB	192	26.1	35.9	6.83
SEM (n = 10)		1.0	0.46	0.61	0.151
Main effect					
Embryonic day					
7		179	26.0	33.4	6.72
11		181	26.3	34.1	6.78
SEM (n = 20)		0.7	0.32	0.42	0.104
Treatment					
PBS		170	26.4	32.0	6.57
HMB		190	25.9	35.5	6.93
SEM (n = 20)		0.7	0.32	0.42	0.104
*P*-value					
Embryonic day		0.109	0.546	0.278	0.679
Treatment		<0.001	0.342	<0.001	0.020
Embryonic × Treatment		0.356	0.931	0.905	0.095

Abbreviations: VH = villus height; CD = crypt depth; VH:CD ratio = villus height to crypt depth ratio; VW = villus width.

1 Each value represents the mean of 10 replicates per each *in ovo* feeding of treatment.

## Discussion

Administration of HMB by *in ovo* feeding on E7 increased BW and chick yield, along with a reduced hatch window, suggesting that HMB may have supported embryonic development and improved hatch synchronization when administered at an earlier embryonic stage. The stronger response observed after *in ovo* feeding of HMB on E7 may be related to the developmental status of Japanese quail embryos at this relatively earlier stage. At this stage, Japanese quail embryos undergo continued tissue differentiation, organ growth, and rapid cellular proliferation, which may increase their responsiveness to exogenous nutritional or bioactive stimuli (Ainsworth et al., 2010). In addition, the proliferation and development of myogenic precursor cells actively occur until approximately embryonic day 12 in Japanese quail (Liu et al., 2023), and primary ossification centers in the long bones also appear around this period (Pourlis et al., 1998), suggesting that *in ovo* feeding prior to this stage may exert more pronounced effects on embryonic development. In previous studies, HMB has been associated with anabolic signaling pathways related to protein metabolism (Kornasio et al., 2009; Girón et al., 2016), which may partly explain the increased BW and chick yield observed in the present study. However, these mechanisms were not directly evaluated, and the present study did not determine the tissue components responsible for the increased BW at hatching. Therefore, this response cannot be specifically attributed to muscle accretion. Moreover, because post-hatch growth was not evaluated, the greater BW at hatch cannot be assumed to translate into sustained growth or production benefits after hatching.

Administration of HMB by *in ovo* feeding on E7 also decreased the hatch window. The hatch window reflects the spread of hatching time among embryos and is commonly used as an indicator of developmental synchrony (Tona et al., 2003). The reduced hatch window may be attributable to greater uniformity in embryonic development, potentially resulting from enhanced energy availability and physiological preparedness for hatching. Previous studies in chickens have reported that *in ovo* administration of nutrient solutions containing HMB, generally in combination with carbohydrates or protein sources, improved hatching BW, glycogen status, intestinal development, and nutrient absorptive capacity, collectively suggesting enhanced physiological preparedness for hatching (Tako et al., 2004; Uni et al., 2005). As hepatic and muscle glycogen concentrations, oxygen consumption, and metabolic rates were not determined in the present study, the physiological mechanisms underlying the reduced hatch window remain to be elucidated. As a main effect of *in ovo* feeding treatment, HMB increased hatch of set, hatch of fertile, BW at hatch, and chick yield, irrespective of the day of administration. These concurrent improvements may reflect enhanced embryonic viability and growth, potentially associated with greater physiological and energetic preparedness for hatching (Kornasio et al., 2009). As embryonic mortality patterns, tissue composition, and energy reserves were not determined in the present study, the mechanisms underlying these responses remain to be elucidated.

HMB administration on E11 resulted in a lower incidence of post-pipping mortality compared with PBS. During the final stage of incubation, embryos experience increased oxygen demand and metabolic activity as they transition from chorioallantoic to pulmonary respiration, a process that requires coordinated hatching muscle activity for successful internal and external pipping (Visschedijk, 1968b; Decuypere and Bruggeman, 2007). During late embryonic development, avian embryos rely heavily on yolk-derived nutrients to sustain rapid metabolic activity (Uni et al., 2005), and hepatic and yolk-sac glycogen reserves have been suggested to serve as important energy substrates during pipping and the transition to pulmonary respiration (Yadgary and Uni, 2012). Although glycogen content and oxygen consumption were not measured in the present study, the reduction in post-pipping mortality may indicate that HMB supported the ability of embryos to complete the hatching process. Late embryonic mortality and post-pipping mortality occur during the final stage of incubation, when embryos experience high metabolic and oxygen demands (De Oliveira et al., 2008). Therefore, the lower incidence of these events in HMB-treated embryos may indicate improved embryonic viability during this critical period.

In the present study, the relative radius length was greater in the HMB group than in the PBS group, whereas the relative tibia length, intestinal length, and chick length were not affected. This finding suggests that the response to HMB was limited to the radius, rather than reflecting a generalized effect on skeletal growth. As individual skeletal elements may differ in their developmental timing and responsiveness during embryogenesis, the increased relative radius length may reflect a site-specific morphometric response (Nowlan et al., 2010). The biological significance of this finding remains to be determined because bone mineralization, mechanical strength, and histological characteristics were not assessed.

The HMB groups had lower relative liver, proventriculus, and intestinal weights than the PBS groups and tended to have lower relative gizzard weights than the PBS groups. A significant interaction was also observed for relative heart weight, with HMB decreasing relative heart weight compared with PBS on E7. However, relative organ weights should be interpreted carefully because they are expressed as percentages of BW. As the HMB group had a greater BW at hatching, the lower relative organ weights may partly reflect a greater overall BW rather than impaired organ development. Relative organ weight does not directly indicate organ function; therefore, absolute organ weight and histological assessments would be required to determine whether organ-specific development was altered. The absence of significant differences in relative breast and thigh weights further supports the interpretation that the increased BW at hatching was not primarily due to muscle-specific accretion. Although a previous study in broilers reported that *in ovo* administration of HMB at E7 via the air cell influenced breast muscle yield (Ma et al., 2020), no such effect on relative breast weight was observed in the present Japanese quail study. This discrepancy may reflect differences in species, dose, developmental stage, or timing of the measurements.

No differences in jejunal morphology were observed between the E7 and E11 groups. During embryonic development, the intestine undergoes stage-specific structural maturation, with villus formation becoming more evident during the later stages of incubation (Uni et al., 2003). As both E7 and E11 occurred before the final phase of intestinal maturation, the difference in injection timing may not have been sufficient to produce distinct jejunal morphological responses at hatching. This may explain the comparable jejunal morphology observed between embryonic days. Regarding the main effects of *in ovo* feeding treatment, the HMB groups showed greater VH, VW, and VH:CD ratios than the PBS groups. Jejunal morphological indices are commonly used to assess the structural development of the intestinal mucosa. Greater VH and VW values may indicate expansion of the mucosal surface area, whereas a higher VH:CD ratio may reflect more advanced structural maturation of the jejunum (Uni et al., 1998). However, functional parameters such as nutrient digestibility, nutrient transporter expression, epithelial barrier function, and tight junction protein expression were not evaluated in the present study. Accordingly, the increases in VH, VW, and VH:CD ratio should be interpreted as structural differences in the jejunal mucosa and do not necessarily indicate improved nutrient absorption, intestinal function, or underlying molecular mechanism.

Several limitations of the present study should be considered. First, HMB was administered via air-cell injection, but the actual timing and extent of embryonic exposure to HMB following injection were not determined. Therefore, potential variability in HMB delivery to the embryo cannot be excluded. Second, the present study primarily evaluated hatching, morphometric, and histological outcomes without functional or mechanistic measurements, including energy metabolism, nutrient utilization, intestinal barrier function, or molecular signaling. Consequently, the observed responses should be interpreted primarily as descriptive changes, and the mechanisms and functional consequences underlying these effects remain to be determined.

## Conclusion

The administration of HMB via *in ovo* feeding may improve hatching performance, reduce late embryonic mortality, and support jejunal structural development in Japanese quail. The effects of HMB appeared to depend partly on the embryonic stage at administration, with E7 administration showing clearer effects on BW at hatching, chick yield, and the hatch window, and E11 administration being associated with reduced post-pipping mortality. As this study primarily evaluated descriptive and morphological endpoints, further studies are needed to determine the underlying mechanisms and functional consequences of *in ovo* HMB administration.

## Author contributions

Ji Won Shin contributed to the study conception and design, experimental work, data collection and analysis, interpretation of the results, and drafting of the manuscript. Geun Yong Park contributed to the study conception and design, experimental work, data collection and analysis, interpretation of the results, and critical revision of the manuscript for important intellectual content. Yeong Bin Kim contributed to the experimental work, data analysis and interpretation, and critical revision of the manuscript for important intellectual content. Ju Yeong Park contributed to the experimental work, data collection and analysis, interpretation of the results, and critical revision of the manuscript for important intellectual content. Jin Ho Jeong contributed to the experimental work and data collection and critically reviewed the manuscript for important intellectual content. Seung Yun Lee contributed to the study conception and design, experimental work, interpretation of the results, supervision, and critical revision of the manuscript for important intellectual content. Jong Hyuk Kim contributed to the study conception and design, experimental work, data collection and interpretation, provision of research resources, supervision, drafting of the manuscript, and critical revision of the manuscript for important intellectual content. All authors reviewed and explicitly approved the final version of the manuscript and agree to be accountable for all aspects of the work, including ensuring that questions related to the accuracy or integrity of any part of the work are appropriately investigated and resolved.

## Disclosures

We declare that we have no financial and personal relationships with other people or organizations that can inappropriately influence our work, and there is no professional or other personal interest of any nature or kind in any product, service and/or company that could be construed as influencing the content of this paper.
